# Modulating Protein Stability to Switch Toxic Protein Function On and Off in Living Cells^1^

**DOI:** 10.1104/pp.18.01215

**Published:** 2019-01-24

**Authors:** Frederik Faden, Stefan Mielke, Nico Dissmeyer

**Affiliations:** a Independent Junior Research Group on Protein Recognition and Degradation, Leibniz Institute of Plant Biochemistry, D-06120 Halle (Saale), Germany; b ScienceCampus Halle, Plant-Based Bioeconomy, D-06120 Halle (Saale), Germany

## Abstract

Toxic proteins are prime targets for molecular farming (the generation of pharmacologically active or biotechnologically usable compounds in plants) and are also efficient tools for targeted cell ablation in genetics, developmental biology, and biotechnology. However, achieving conditional activity of cytotoxins and maintaining the toxin-expressing plants as stably transformed lines remain challenging. Here, we produce a switchable version of the highly cytotoxic bacterial RNase barnase by fusing the protein to a portable protein degradation cassette, the low-temperature degron cassette. This method allows conditional genetics based on conditional protein degradation via the N-end rule or N-degron pathway and has been used to vice versa accumulate and/or deplete a diverse variety of highly active, unstable or stable target proteins in different living multicellular organisms and cell systems. Moreover, we expressed the barnase fusion under control of the trichome-specific *TRIPTYCHON* promoter. This enabled efficient temperature-dependent control of protein accumulation in Arabidopsis (*Arabidopsis thaliana*) leaf hairs (trichomes). By tuning the levels of the protein, we were able to control the fate of trichomes in vivo. The on-demand formation of trichomes through manipulating the balance between stabilization and destabilization of barnase provides proof of concept for a robust and powerful tool for conditional switchable cell arrest. We present this tool as a potential strategy for the manufacture and accumulation of cytotoxic proteins and toxic high-value products in plants or for conditional genetic cell ablation.

## Abstract (precis)

Conditional toxicity can be switched on/off to arrest individual cells in vivo by a portable protein degradation cassette fused to a highly toxic protein.

## Conditional Modulation of Protein Levels in Multicellular Organisms

The low-temperature degron cassette (lt-degron) fused to a protein of interest (POI) can efficiently control accumulation or degradation of diverse target proteins in a temperature-dependent manner in vivo in multicellular organisms (Dissmeyer and Schnittger, 2016; Faden et al., 2016b; Dissmeyer, 2017). The technique is based on altering the in vivo stability of the lt-degron:POI fusion protein by using a temperature stimulus (Dohmen et al., 1994; Faden et al., 2014) and relies on the conditional degradation of the entire fusion by the N-end rule pathway of protein degradation (Dissmeyer et al., 2018; Dissmeyer, 2019; Varshavsky, 2019) that leads to fast removal from the cell.

The lt-degron technology itself is based on a combination of the Ubiquitin (Ub) fusion technique (Bachmair et al., 1986; Baker et al., 1992; Naumann et al., 2016) and temperature-sensitive destabilization of fusions to a dummy protein, mouse dihydrofolate reductase (DHFR). It was originally introduced in the budding yeast *Saccharomyces cerevisiae* (Dohmen et al., 1995). The Ub fusion technique allows users to deliberately expose N-terminal amino acids of choice in vivo by fusing the 76-residue Ub moiety N terminally of the desired residue in the lt-degron, either Phe or Arg, both primary destabilizing residues according to the N-end rule. Ub is cotranslationally cleaved off by deubiquitinating enzymes (Ub-specific proteases). According to the current understanding, either early conformational changes of surface-exposed Lys residues that are made available as polyubiquitination acceptor sites by E2-E3 Ub ligase complexes were suggested as the mechanism for conditionality or, much later, conditional degradation by the proteasomal pathway, where polyubiquitination mediates protein degradation, as the rate-limiting step (Faden et al., 2016b).

The lt-degron:POI fusion can thus efficiently accumulate at a permissive temperature or can be removed from the cell at a restrictive temperature, generating an artificial temperature-sensitive variant of the POI (Dissmeyer and Schnittger, 2016; Faden et al., 2016b; Dissmeyer, 2017). In contrast to many other systems for conditional protein expression and gene regulation (for review, see Faden et al., 2014; Yu et al., 2015; Natsume and Kanemaki, 2017; Trauth et al., 2019), the lt-degron approach relies on posttranslational interference with protein stability by degradation of the entire fusion protein and is applicable in living multicellular organisms in both plants and animals.

## The LT-Degron Works Via Shifting Between Elevated and Reduced Ambient Temperatures

Plant growth and development are clearly influenced by temperature (Gray et al., 1998; Larkindale et al., 2005; Koini et al., 2009; Kumar and Wigge, 2010; Franklin et al., 2011; for review, see Quint et al., 2016), and clear phenotypic differences will be generated when comparing plants constantly grown under low (permissive) or high (restrictive) temperatures. Notably, these are near but not above the lower or upper ends (approximately 4°C and 38°C) of the physiological temperature range of Arabidopsis (*Arabidopsis thaliana*; Larkindale et al., 2005; Jung et al., 2012). Cold stress includes chilling (0°C–15°C) and freezing (less than 0°C) temperatures (Chinnusamy et al., 2007; Zhu et al., 2007). Altered thermomorphogenesis, including effects of the plant high-temperature adaptation responses like hypocotyl and petiole elongation, increased leaf size, and earlier flowering, must be taken into consideration. Moreover, genotype and accession affect the phenotype in Arabidopsis depending on the ambient temperature (Ibañez et al., 2017). Nonetheless, the lt-degron offers tight control over the levels of active/functional protein that directly correlates to the temperature stimulus used to induce the system.

## Conditional Protein Expression in Molecular Farming

Molecular farming, the generation of pharmacologically active or biotechnologically usable compounds in plants, is an emerging topic especially for the production of peptides and proteins requiring special glycosylation patterns impossible to generate in classical fermenter-based expression systems using microorganisms like bacteria (Stoger et al., 2014). Various cytotoxic peptides are under investigation as highly potent cancer therapeutics (Boohaker et al., 2012). Some toxic proteins, such as the mistletoe lectins, both from Korean mistletoe (*Viscum album coloratum*) and European mistletoe (*Viscum album* [Loranthaceae]), possess anticancer properties when administered orally (Pryme et al., 2007), and recombinantly produced immunotoxins are in clinical and preclinical evaluation for cancer treatments (Kelly et al., 2012; Madhumathi J and Verma, 2012). So far, biologically active lectin proteins from plants and animals could mainly be produced in *Escherichia coli*, albeit with moderate yields (Lam and Ng, 2011).

The production of toxic proteins in plants is still challenging, mainly due to difficulties in transgene maintenance. So far, production of phytotoxic peptides, such as antimicrobial peptides, has been difficult and mostly needs either further modification of the peptide (Company et al., 2014b) or was achieved through vigorous control of transgene expression through inducible promoters (Company et al., 2014a). The lt-degron technique offers tremendous advantages over inducible promoters due to easier control and nonaversive effects of the stimulus of elevated ambient temperature versus chilling.

## RNase as a Test Case for Switchable Expression of Toxic Proteins

The bacterial RNase barnase (BAR), a potent, nonspecific RNase secreted by the soil bacterium *Bacillus amyloliquefaciens* (Buckle and Fersht, 1994), possesses anticancer properties (Edelweiss et al., 2008) and has also been used as a potent tool for plant breeders to create male-sterile mutants in oilseed rape (*Brassica napus*; Mariani et al., 1992), tobacco (*Nicotiana tabacum*), tomato (*Solanum lycopersicum*), and pepper (*Capsicum annuum*; Burgess et al., 2002), *Nicotiana benthamiana*, Arabidopsis, and wheat (*Triticum* spp.; Gils et al., 2008). Additionally, it has been exploited as a cell ablation tool to destroy inflorescences to prevent the spread of transgenes from birch (*Betula* spp.), tobacco, and Arabidopsis (Lännenpää et al., 2005). Other applications include a system to defend potato (*Solanum tuberosum*) plants against the pathogen *Phytophthora infestans* (Strittmatter et al., 1995) and a conditional cell ablation tool in mammalian cell culture (Leuchtenberger et al., 2001). Due to the conserved mechanism of BAR activity as a nonspecific RNase, it is considered to be highly cytotoxic in literally all organisms, highlighting the broad spectrum of possible applications for a controllable RNase.

## Toxic Proteins as a Genetic Tool to Destroy Living Cells In Situ

Cell ablation is a powerful tool to study the effects of elimination of entire cell populations in a biological context. Conditional as well as constitutive techniques have been extensively used in the animal field (e.g. to map cell populations in the murine nervous system, where the natural resistance of some mouse cell types against the highly cytotoxic *Diphtheria* toxin chain A [DT-A] has been exploited). By controlling the expression of a DT-A receptor, cells were indirectly but efficiently ablated upon exogenous administering of DT-A (Saito et al., 2001). DT-A was also used extensively to induce cell death in yeast, fruit flies (*Drosophila melanogaster*), and plants (Bellen et al., 1992; Guerineau et al., 2003). Another example is a regeneration study in zebrafish (*Danio rerio*) through the guided, enzymatic conversion of a prodrug (precursor drug) into an active, DNA-toxic compound by a bacterial nitroreductase (Curado et al., 2007). Another system, albeit not applicable in plants, is the auxin-controlled suicide module based on rapid degradation of the inhibitory chaperone ICAD (inhibitor of caspase-activated DNase). Its degradation enabled caspase activity inducing cell death (Samejima et al., 2014).

Cell ablation by toxic proteins needs to be tightly controlled in a conditional rather than constitutive manner. Otherwise, the host organism or expressing tissue will be irreversibly destroyed. However, most conditional, nonconstitutive, cell ablation protocols rely on the addition of exogenous compounds such as hormones or other small molecules triggering cell death. Depending on the experimental conditions, these systems struggle with uneven induction of the system, low penetration, or metabolic secondary effects of the inducing agents. Moreover, the systems have intrinsic delays in response due to the nature of the induction of transcription followed by translation of the target protein. The protein accumulation is irreversible, which makes control at the level of acute toxicity, namely the protein level, impossible.

Here we report a strategy to control BAR protein levels via temperature and therefore turn its activity on or off. This allows generation of a phenotype on demand and results in conditional organ formation versus ablation in intact, living plants. We combined the lt-degron with the cytotoxic BAR protein and expressed the resulting lt-BAR construct in leaf hairs (trichomes), which are specialized single cells of the epidermis, under control of the trichome-specific *TRIPTYCHON* (*TRY*) promoter (Pesch and Hülskamp, 2011). This setup allowed us to control trichome cellular fate in a temperature-dependent manner and serves as a proof of concept for conditional cell ablation. Moreover, this tool paves the way toward more efficient molecular farming of cytotoxic proteins in planta.

## RESULTS

### Conditional Toxicity of RNase Leads to Specific Cell Ablation in Stable Transgenic Lines

The lt-degron approach requires an N-terminal fusion of the portable degron cassette to the POI that will render the POI temperature sensitive (Faden et al., 2014, 2016b; Dissmeyer, 2017). For this cassette, we used a permissive temperature of 14°C, which allows the POI to remain stable, and a restrictive temperature of 28°C, which induces degradation of the POI. Therefore, the lt-degron cassette was fused to the coding sequence of BAR that contains an artificial intron, efficiently suppressing the expression of functional toxic protein in bacteria due to possible promoter leakiness (Gils et al., 2008).

**Figure 1.**
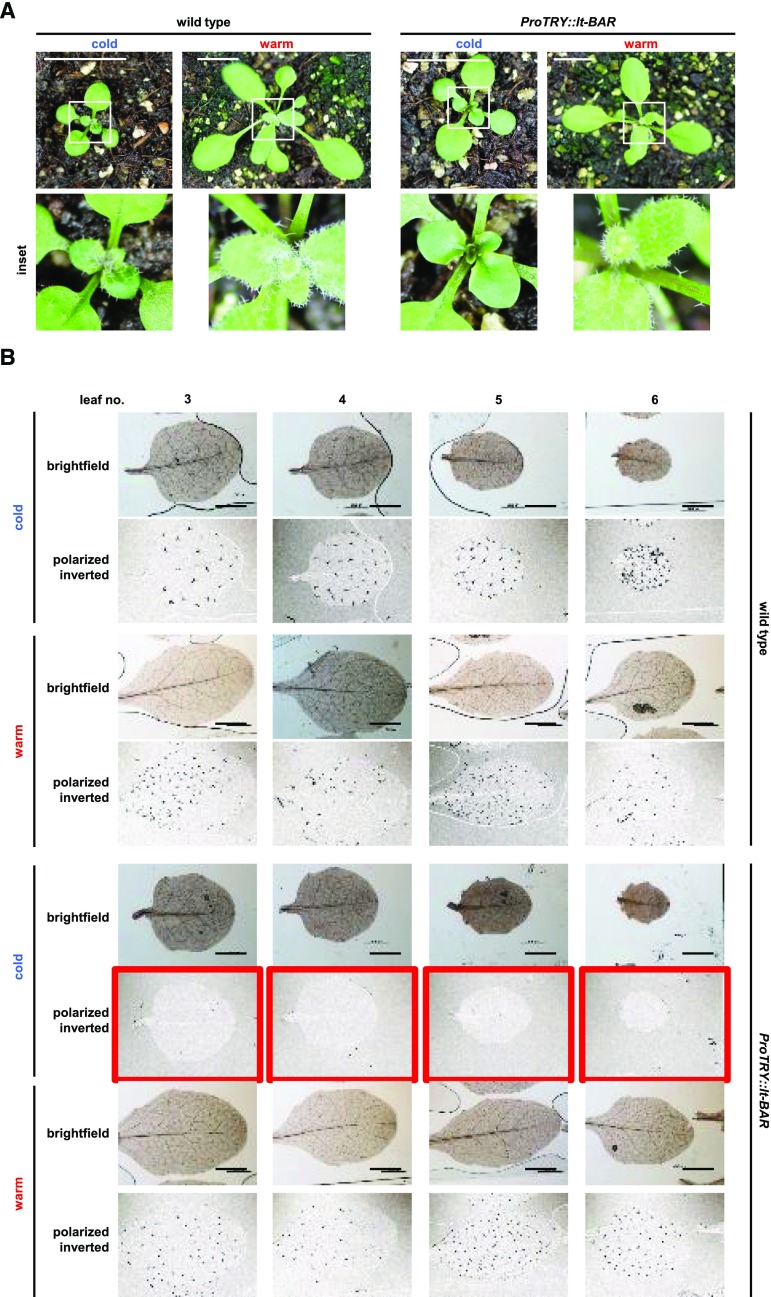
Conditional function of lt-BAR mediates robust temperature-dependent ablation of specific cells. A, Plants expressing *ProTRY*::*lt-BAR* and the wild-type grown for 2 weeks at permissive (cold) and restrictive (warm) temperatures. Leaves of transgenic plants appear glabrous at the permissive temperature. Bars = 1 cm. B, In-depth analysis of true leaves of *ProTRY*::*lt-BAR* and wild-type plants using polarized light microscopy. Conditionally cold- or warm-grown plants are shown 3 weeks after germination. Cold-grown transgenic plants show glabrous leaves (red boxes). Bars = 500 μm.

When expressed in trichomes of wild-type Arabidopsis plants under the control of the *TRY* promoter (*ProTRY*), lt-BAR resulted in efficient ablation of trichomes. When plants were grown at the permissive temperature of 14°C, leaves appeared completely glabrous. Constitutive growth under the restrictive temperature of 28°C led to the opposite effect, namely trichomes formed on newly emerging leaves (Fig. 1A). Next, we evaluated the efficiency of the organ ablation through analysis of trichomes within the leaf epidermis. We used polarized light microscopy to analyze trichome spacing and number on the leaves. The wild type developed trichomes under both permissive and restrictive conditions, whereas no trichomes could be observed on mature leaves of *ProTRY*::*lt-BAR* plants when grown at the permissive temperature (Fig. 1B). In contrast, these *lt-BAR*-expressing plants fully retained trichomes when grown under restrictive conditions. At the restrictive temperature, leaves of *lt-BAR*-expressing plants were indistinguishable from wild-type plants (Fig. 1B).

### Transient Expression of lt-BAR in *N. benthamiana* Leaves

**Figure 2.**
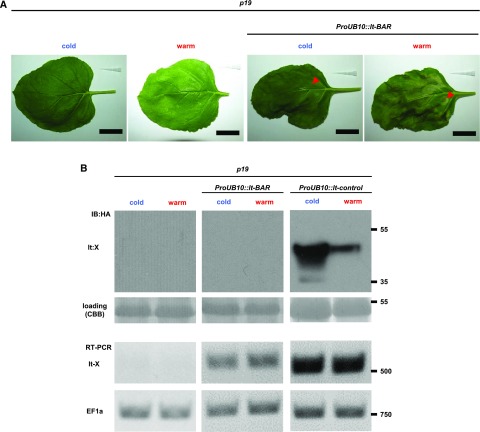
Transient expression of lt-BAR and transcript analysis. A, Transient expression of lt-BAR in leaves of *N. benthamiana* leads to a temperature-independent chlorosis/necrosis phenotype. Control plants were transformed with agrobacteria carrying the p19 plasmid only. Red arrowheads indicate observed necrosis on infiltrated leaf areas 3 d after temperature shift. Bars = 3 cm. B, Analysis of *lt-BAR* transcript and protein levels demonstrates that it is expressed but protein amounts are below the detection limit. An lt-control protein (Bcl2-associated X protein [lt:X]) expressed from the same promoter served as a positive control during western-blot analysis. Control plants were transformed with agrobacteria carrying the p19 plasmid only. CBB, Coomassie Brilliant Blue G250; HA, hemagglutinin; IB, immunoblot; RT, reverse transcription.

The lt-degron, as a conditional protein shut-off technique, relies on a temperature-sensitive switch between POI depletion and accumulation at the protein level without altering transcript levels (Faden et al., 2014, 2016b; Dissmeyer, 2017). Therefore, we tested conditional accumulation of lt-BAR protein upon a shift of *ProTRY*::*lt-BAR*-expressing Arabidopsis plants to permissive temperature. Although the cell ablation phenotype was visible (Fig. 1), detection of lt-BAR protein with standard western-blotting techniques gave only variable results. Because detection of *lt-BAR* transcripts from *ProTRY*::*lt-BAR* plants by reverse transcription PCR proved to be difficult, we transiently expressed the lt-BAR construct under the control of the *UBIQUITIN10* promoter (*ProUBQ10*::*lt-BAR*) in *N. benthamiana* leaves. The expression led to a broadly temperature-independent severe phenotype with developing necrosis and chlorosis of the leaves at both permissive and restrictive temperatures. The phenotype appeared to be stronger at the restrictive temperature (Fig. 2A). We tried once more to demonstrate the connection between the phenotype and the abundance of lt-BAR fusion protein by western blotting and reverse transcription PCR. Here, we could obtain transcript data clearly linking the observed phenotype to the expression of lt-BAR (Fig. 2B).

### Conditional Toxin Function Can Be Shifted and Is Active at Ambient Temperature

**Figure 3.**
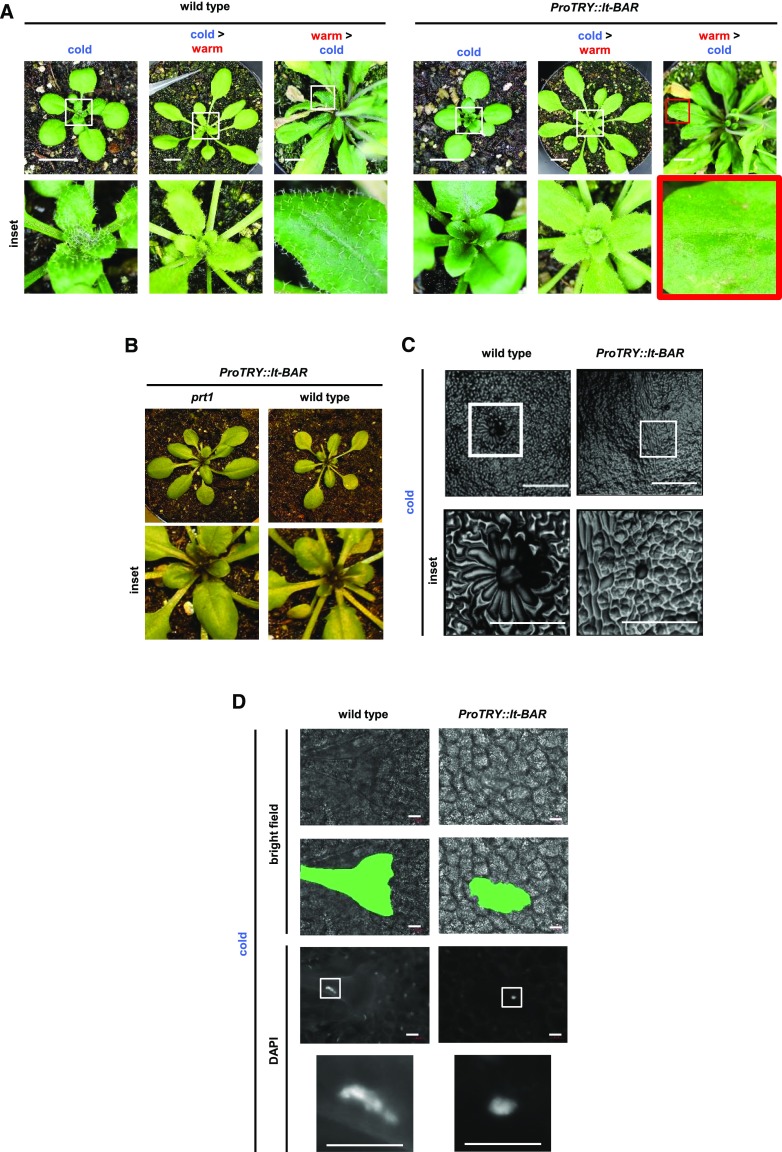
The phenotype caused by lt-BAR follows the temperature stimulus but drives cells into growth arrest rather than cell death. A, Plants grown at the permissive temperature were shifted to the restrictive temperature after 2 weeks and shifted back to the permissive temperature after another 9 d. The glabrous leaf phenotype occurs in relation to the temperature stimulus. Bars = 1 cm. B, *ProTRY*::*lt-BAR* elicits a reliable on phenotype under standard greenhouse conditions in 21-d-old plants showing glabrous leaves. C, Agarose imprints of the leaves of wild-type and *ProTRY*::*lt-BAR* plants reveal that expression of lt-BAR impairs trichome-initiating cells in developing the typical single-celled trichome structure within the plant tissue. Bars = 500 μm. D, Bright-field image and DAPI staining of leaf epidermis. The green marks in the second row show the outline of the trichome-forming cell. The unexpected shape in the wild type results from the mature trichome being pushed on the surface of the leaf by the cover slide. Bars = 50 μm.

One advantage of the lt-degron system is its tunability and reversibility depending on both time of action and temperatures. First, the sensitivity of the lt-BAR system was determined by growing lt-BAR plants at the permissive temperature for 3 weeks, followed by shifting them to the restrictive temperature for 9 d, and finally returning them back to the permissive temperature. During this process, trichomes on freshly developed leaves were monitored. The trichome phenotype of shifted plants followed the temperature stimulus, exhibiting glabrous leaves at the permissive temperature, developing wild-type-like leaves containing trichomes when grown under the restrictive temperature, and again showing glabrous leaves when shifted back to the permissive temperature (Fig. 3A). This shows that the function of lt-BAR follows the temperature stimulus exhibiting efficient on/off transitions as previously described for the lt-degron for nontoxic proteins (Faden et al., 2016b; Dissmeyer, 2017).

To estimate temperature ranges where phenotypic responses were likely to occur and to find out if the system was leaky under standard growth conditions, the phenotype was determined at room temperature. Therefore, we asked whether lt-BAR-expressing plants could successfully elicit a glabrous leaf phenotype when grown at standard greenhouse conditions and found, indeed, that lt-BAR was able to robustly cause glabrous leaves under these conditions (Fig. 3B).

### The Conditional Toxin Causes Growth Arrest Rather Than Cell Death

In vertebrate and yeast cells, a cell suicide module made use of ectopic activation of the nuclease CAD mediated by the auxin-inducible degron system. Here, the activation was accomplished by adding the plant hormone auxin to the cell cultures relying on auxin-induced rapid and conditional degradation of ICAD. This activated CAD and triggered apoptotic DNA fragmentation and chromatin condensation. This indicated that ectopic activation of caspases can be used in nonapoptotic living cells to induce cell death (Samejima et al., 2014).

In our case, we tested the effect of lt-BAR on trichome development to find out if we can also induce programmed cell death in a multicellular organism analogous to the cell suicide module that worked in single cells. Trichome spacing and TRY activity begin in the leaf primordia (Larkin et al., 1996). TRY, as the first identified negative regulator of trichome development, is not important for the actual formation of the trichome itself but is crucial for both spacing and patterning (Hülskamp et al., 1994). In this context, TRY suppresses trichome initiation in neighboring cells (Schnittger et al., 1999; Digiuni et al., 2008; Pesch et al., 2013). Therefore, ablation or arrest of a TRY-expressing cell could allow TRY expression in the neighboring cell, potentially resulting in its own ablation or arrest, starting a chain reaction in the living tissue. To address this hypothesis, we prepared agarose imprints of the leaf surface of lt-BAR plants grown at the permissive temperature. We found that the trichome-forming cells went into an early arrest and were not able to form the typical trichome structure (Fig. 3C). To test whether the cells underwent cell death or resided in a state of growth arrest, nuclei were fluorescently stained with the DNA stain 4',6-diamino-phenylindole (DAPI). All cells showed normal nuclei, indicating cell arrest rather than cell death (Fig. 3D).

**Figure 4.**
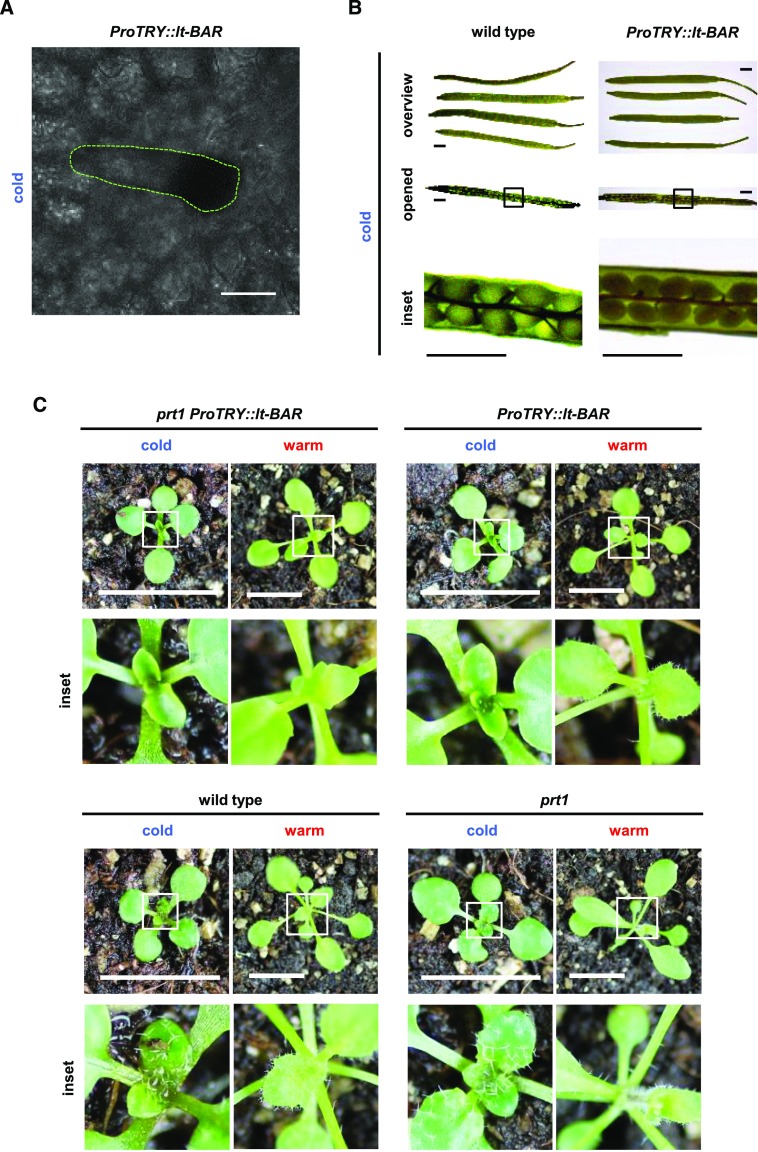
Phenotypes of lt-BAR and requirement of E3 ligase PRT1 for functionality. A, Example of a trichome precursor (green-dotted shape) forming on a late leaf of a 5-week-old plant expressing *ProTRY*::*lt-BAR* and grown at permissive conditions. Bar = 50 μm. B, Siliques of wild-type and *ProTRY*::*lt-BAR* plants. Bars = 1 mm. C, Expression of lt-BAR in the *prt1* mutant background causes temperature-independent cytotoxic effects and constitutive phenotypes of trichome ablation. Bars = 1 cm.

In very rare cases, cells were able to partially overcome the, presumably, toxic effect of lt-BAR. This was indicated by started initiation and formation of a trichome in these cells (Fig. 4A). However, mature trichomes were never found on any lt-BAR-expressing plant at the permissive temperature. The lt-BAR-expressing line showed arrested cells that were able to grow out into basic trichome precursors (Figs. 3, C and D, and 4A).

TRY is expressed in seeds (compare expression data from the Arabidopsis eFP-browser bar.utoronto.ca/efp/cgi-bin/efpWeb.cgi; Winter et al., 2007), and therefore, seed development might be affected in *ProTRY*::*lt-BAR* plants. However, when plants were grown under the permissive temperature, siliques appeared indistinguishable from wild-type plants (Fig. 4B) and seeds did not show any alterations in number, shape, or germination rate.

### The N-End Rule E3 Ub Ligase PROTEOLYSIS1 Is Required for Modulating lt-BAR Toxicity

We recently showed that in Arabidopsis, lt-degron fusions, for technical reasons, depend on the presence of the N-end rule E3 Ub ligase PROTEOLYSIS1 (PRT1; Faden et al., 2016b), which has a high preference for aromatic N-terminal amino acids (Potuschak et al., 1998; Stary et al., 2003; Naumann et al., 2016; Dong et al., 2017; Reichman and Dissmeyer, 2017; Faden et al., 2018; Mot et al., 2018). To generate a stable genetic condition with constitutively stabilized lt-BAR and to test whether the observed phenotype was a result of the fusion to the lt-degron cassette or rather a response of BAR activity to the changed temperature, we introgressed *ProTRY*::*lt-BAR* into the *prt1* mutant. Indeed, lt-BAR in the *prt1* mutant background resulted in a complete and temperature-independent stabilization of the glabrous phenotype, where plants are not able to produce normally developed and spaced trichomes on the leaf surface (Fig. 4C).

## DISCUSSION

### Function of the Toxic Protein Depends on the Ambient Temperature and Is Switchable

We have shown that, by using a fusion of the lt-degron to the RNase BAR, we can turn on/off its cytotoxicity and thus ablate cells in living plants. When plants were grown at the permissive temperature, leaves appeared completely glabrous, as has been observed from plant mutants for any of the transcriptional regulators required for trichome initiation and formation, like the positive regulators *TRANSPARENT TESTA GLABRA1*, *GLABRA1*, and *GLABRA3* (Schnittger et al., 1998; Bouyer et al., 2008; for review, see Schnittger et al., 1996; Schnittger and Hülskamp, 2002; Hülskamp, 2004). In contrast, the *lt-BAR*-expressing plants fully retained trichomes when grown under the restrictive condition. This demonstrates the efficiency and temperature sensitivity of the toxicity and, therefore, the molecular function of the lt-BAR fusion protein as well as its effect on cell fate and organ formation. At the restrictive temperature, leaves of *lt-BAR*-expressing plants were indistinguishable from the wild type, demonstrating an efficient elimination of the toxic function of the lt-BAR fusion protein. Apparently, this temperature-dependent protein (function) shut off was successful to an extent, where the transcribed *lt-BAR* was unable to elicit a glabrous phenotype. Whether the control occurs at the posttranslational level, as expected from conditional degradation mediated via the PRT1/N-end rule pathway, is not clear at this point, as immunoblots produced only variable results. It remains to be shown that the actual protein level was controlled.

Expression of lt-BAR in *N. benthamiana* led to a temperature-independent phenotype that appeared to be more pronounced at the restrictive temperature (chlorosis, necrosis). This could be explained by a possible override of the N-end rule degradation system caused by expression under control of the strong *UBQ10* promoter combined with a possibly higher nuclease activity of the lt-BAR protein at the higher temperature.

### Levels of Cytotoxic Protein Remain Undetectable But Can Successfully Eliminate Cells

Whether this loss of cell arrest relied on degradation of the actual lt-BAR fusion protein that was similarly shown for many other target proteins destabilized by the classical temperature-dependent-method in yeast or cell culture (Dohmen et al., 1994; Su et al., 2008; Bernal and Venkitaraman, 2011) and living multicellular organisms (Faden et al., 2016b) remains to be shown. We extensively tried to reliably determine the chimeric protein via western blot from stably transformed Arabidopsis plants and from leaf tissue of transiently transformed *N. benthamiana* plants. However, reproducible western-blotting results could not be obtained, although a highly efficient triple HA tag was used as an epitope together with a highly specific antibody (HA.11, clone 16B12). At the same time, detection of *lt-BAR* transcripts gave variable results when samples from stably transformed Arabidopsis plants were used. This could be due to very low levels of transcript as a combinatorial effect of low promoter activity (ProTRY), additional RNase activity and therefore high toxicity of the BAR fusion, and very low amounts of expressing trichome cells in the tissue sample that also contained the rest of the leaf material.

However, in the background of the N-end rule-deficient *prt1* mutant that we used as a control, the temperature-dependent cell arrest effect was eliminated, suggesting the involvement of protein degradation via the Ub proteasome system, which is known to be responsible for the protein turnover mechanism of the lt-degron approach (Faden et al., 2016b). This is also in line with the ubiquitination activity and specificity of the E3 Ub ligase PRT1 as demonstrated previously (Mot et al., 2018). Therefore, it is likely that the effect of lt-BAR relies on targeted protein degradation that was responsible for the temperature-responsive phenotype.

### Boosting Levels of Toxic Proteins

To increase the amounts of protein produced, two strategies have been suggested, namely the use of ubiquitous promoters and of trichome-overproducing Arabidopsis mutants. Both strategies will be further tested to document and improve the conditional expression of proteinaceous toxins. One approach was to express lt-BAR under the control of promoters like *UBQ10* and *CDKA;1* (Nowack et al., 2007; Harashima et al., 2016) and increase the number of trichomes by using higher order mutants that lack TRY function and also that of the negative regulator of nonroot hair cells, CAPRICE (CPC; Schellmann et al., 2002), and ENHANCER OF TRY AND CPC1, which acts redundantly with TRY and CPC in trichome and root hair cell patterning (Kirik et al., 2004). Detection of an accumulation of (probably) minute levels of cytotoxic protein(s) will be required to fine-tune the lt-BAR system for genetic studies and identification of potential biotechnological approaches. Therefore, finding the ideal sampling time points and using highly sensitive protein detection methods (Faden et al., 2016a) could be useful.

### Stability of the Conditional Toxin Function under Variable Temperatures

For optimal use, the minimal restrictive (upper level) and maximal permissive temperature (lower level) should be determined. A starting point is the observation of the phenotype under standard growth conditions where lt-BAR-expressing plants produced a glabrous leaf phenotype. Functionality of lt-BAR at ambient temperature could facilitate its application and reduce the need for controlled growth facilities maintained at the restrictive conditions. Our experiments under standard greenhouse conditions suggested sufficient stability of the fusion protein even at a temperature higher than the permissive temperature, in our study set to 14°C. The lt-BAR-expressing plants maintain the glabrous leaf phenotype under greenhouse conditions even though this environment represents a significantly less controlled environment, with relatively high temperature fluctuations compared with the permissive temperature used in the growth cabinets. This highlights that permissive temperatures need to be determined case by case and are not necessarily bound to temperatures as low as, for example, 14°C as in this study or higher as shown previously (Faden et al., 2016b). However, these relatively low growth temperatures represent safe growth conditions where the lt-BAR system and previously tested constructs reliably produce molecular or developmental phenotypes.

Moreover, intensity and timing of the cell death strongly depend on the genetic background and proteolytic setting of the target organism and must be taken into account (Havé et al., 2018). Then, the N-end rule pathway is of particular importance in the molecular response to various environmental stresses (White et al., 2017; Dissmeyer, 2019; Vicente et al., 2019). Therefore, functionality of the protein degradation system irrevocably linked to the lt-degron might perform variably depending on the growth conditions of the host plants.

### Growth Arrest Versus Cell Death

lt-BAR plants grown at the permissive temperature went into an early arrest but did not collapse or deteriorate. They rather remained within the cellular context, clearly indicating that cell death was not executed in this setup. This could be explained by the *TRY* promoter being active for a limited time only, which allowed the expression of lt-BAR only at low levels. These could have been sufficient to arrest cell growth but not to trigger cell death. Another possibility is that an equilibrium was reached where synthesis of active lt-BAR and turnover via the degron leading to full inactivation was reached. Similar degradation at the permissive temperature has been demonstrated previously for an lt-degron-TTG1 protein fusion (Faden et al., 2016b). Also, growth arrest of a single cell, including its functional disruption, does not seem to trigger trichome formation in neighboring cells, indicating that trichome spacing is a restricted process only happening for a defined period during leaf formation. Then, in rare cases, cells were able to partially overcome the presumably toxic effect of lt-BAR, indicated by started initiation and formation of a trichome in these cells. This indicates that indeed the *TRY* promoter activity was reduced and that cells were possibly able to degrade lt-BAR over an extended period of time even at the permissive temperature.

### Complete Elimination of Mature Trichomes by a Switchable Nuclease

It is noteworthy that no mature trichomes could be found on lt-BAR-expressing plants at the permissive temperature. The phenotype elicited by lt-BAR strikingly resembled the *glabra2* (*gl2*) mutant, which lacks a functional copy of the transcription factor GL2 (Rerie et al., 1994; Schwab et al., 2000). The lt-BAR line showed similarly arrested cells that could grow out into basic trichome precursors as described for the *gl2* mutant. GL2 is thought to be mainly important for trichome formation; however, a role in trichome patterning was also discussed (Rerie et al., 1994; Hülskamp, 2004). Our results at the cellular level also shed new light on the activity of the *TRY* promoter, because it controls the expression of the cytotoxic nuclease that, in turn, precisely leads to arrest of these very cells with TRY promoter activity. This adds new data to previously described TRY promoter activity determined using classical *ProTRY*::*GUS* fusions used as transcriptional reporters (Schellmann et al., 2002; Digiuni et al., 2008; Pesch et al., 2013).

### Comparing with Other Cell Suicide Systems

The lt-BAR system might be more advantageous compared with other conditional and/or inducible systems used to date. A detailed molecular description of the lt-BAR cellular suicide module, especially of the actual protein accumulation versus degradation, remains open and requires further studies. Control over the lt-BAR protein is easy to execute, namely by temperature. Therefore, stabilization as well as degradation and tuning of active protein are easy to carry out. Additionally, exogenous addition of compounds and/or other stimuli for induction of the system are not needed, hence eliminating issues connected to infiltration, uneven induction of the system due to uneven perfusion of inducing agents, as well as possible toxic side effects on the system by the compounds and solvents used. Additionally, the temperature stimulus is easily controllable, enabling easy tuning and regulation of the system and therefore rendering maintenance of the lt-BAR transgene simple and straightforward.

## CONCLUSION AND PERSPECTIVES

We have shown that lt-BAR can be used as a switchable toxin to efficiently mediate cell arrest and thereby influence organ fate in Arabidopsis. In our system, BAR appears to be extremely toxic. Therefore, only very small amounts may be needed to provoke a phenotype. This makes it an excellent tool for cell ablation studies.

Too-low permissive and too-high restrictive temperatures, if applied for periods of time where regular temperature effects become visible in the wild type, can complicate developmental comparisons in cell ablation studies. Untransformed plants need to be grown in parallel as controls (Dissmeyer, 2017). It is advised to apply the trigger temperatures only for short times, from minutes to hours, to generate pulses of protein as shown previously (Faden et al., 2016b), thereby reducing the impact of longer growth periods at either cold or warm temperatures.

lt-BAR could be used under control of various promoters, analogous to studies implementing promoter-GUS or promoter-GFP reporter lines to determine spatiotemporal resolutions of promoter activity. Such an approach would benefit from highly localized and conditional toxicity to mark cells with active promoters on a cellular level in vivo. Promoter-GUS signals are often hard to interpret, and the toxic lt-BAR module might represent a good additional tool for promoter analysis. It will be possible to use lt-BAR to dissect the timing of promoter activity and elucidate the general requirement of the cells or cell lineages where the tested promoters are active. lt-BAR-expressing cells will be significantly disturbed in their growth and differentiation, as seen here. Thus, our tool enables researchers to take views from different angles (e.g. in the context of trichome development) when used under control of various trichome promoters for which, so far, only transcriptional GUS reporters could be used.

Taken together, the suitability of the lt-degron system for expressing toxic proteins in the frame of preparatory approaches for molecular farming was not comprehensively demonstrated in this study, but we present a highly versatile and simple system for conditional cell and organ ablation.

## MATERIALS AND METHODS

### Cloning and DNA Work

Constructs were generated by fusion PCR using *Pfu* polymerase (Nørholm, 2010), and amplicons were purified with ExoSap-IT (USB) preceding fusion PCR. All fusions were flanked by Gateway *attB1/attB2* sites and recombined by BP reactions into *pDONR201* (Invitrogen). The lt-degron cassette (K2) that is extensively described elsewhere (Faden et al., 2016b; Dissmeyer, 2017) was amplified from a Gateway entry clone containing *K2*:*TTG1* (Faden et al., 2016b) with TMV_att_frw (5′-GGG​GAC​AAG​TTT​GTA​CAA​AAA​AGC​AGG​CTT​ACT​CGA​GCT​GCA​GAA​TTA​CTA​TTT​ACA​ATT​AC-3′) and K2barn_f1_rev (5′-TGT​GCC​ATA​GCA​CCA​GCA​CCA​GCG​TAA-3′). The lt-degron K2 contains a triplet for the destabilizing bulky and hydrophobic amino acid Phe at the Ub-DHFR junction that gets exposed as a neo-N terminus (Perrar et al., 2019). Phe is used as a destabilizing N-terminal residue because it was shown to be recognized by the Arabidopsis (*Arabidopsis thaliana*) N-end rule pathway (Bachmair et al., 1993; Potuschak et al., 1998; Stary et al., 2003; Faden et al., 2016b; Mot et al., 2018; Dissmeyer, 2019; Dissmeyer et al., 2019). The lt-degron module is publicly available as Gateway-compatible *pEN-L1-K2-L2* (Addgene ID 80684) for modular cloning.

BAR, including the artificial intron, was amplified from *pICH43601* (a kind gift of Sylvestre Marillonnet, Leibniz Institute of Plant Biochemistry) using primers K2barn_f2_frw (5′-CTG​GTG​CTA​TGG​CAC​AGG​TTA​TCA​ACA​CGT​T-3′) and K2barn_f2_rev (5′-GGG​GAC​CAC​TTT​GTA​CAA​GAA​AGC​TGG​GTA​TTA​TCT​GAT​TTT​TGT​A-3′). A fusion PCR was carried out using primers TMV_att_frw and K2barn_f2_rev, leading to BP-compatible Gateway recombination sites. After subcloning into *pDONR201* by BP reaction, the resulting *pENTR*::*lt-BAR* entry clones were sequenced and recombined by LR reaction into the *attR* site-containing binary Gateway destination vector *pAM-PAT*:*ProTRY*:*GW* (Addgene ID 79755; Pesch and Hülskamp, 2011), yielding *pAM-PAT*:*ProTRY*::*lt-BAR* for generation of stable transgenic plants. These Gateway-compatible destination vectors are derived from *pAM-PAT-MCS* (multiple cloning site; GenBank accession no. AY436765), derivative of *pPAM* (GenBank accession no. AY027531; Rademacher et al., 2002), and carry the *Streptomyces hygroscopicus bar* gene that translates to phosphinothricin-*N*-acetyltransferase (PAT) as a plant selection marker conferring resistance against dl-phosphinotricin. This vector was used for *Agrobacterium tumefaciens*-mediated transformation of Columbia-0 wild-type Arabidopsis plants.

### Plant Work and Growth Conditions

Arabidopsis plants were grown either on a steamed (sterilized for 3 h at 90°C) soil mixture consisting of Einheitserde Classic Kokos (45% [w/w] white peat, 20% [w/w] clay, 15% [w/w] block peat, and 20% [w/w] coco fibers [10-00800-40; Einheitserde Werkverband]), 25% (w/w) vermiculite (grain size, 2–3 mm [29.060220; Gärtnereibedarf Kamlott]), and 300 to 400 g of Exemptor (100 g kg^−1^ thiacloprid [802288; Hermann Meyer]) per m^3^ of soil mixture or aseptically in vitro on plastic petri dishes containing one-half-strength Murashige and Skoog (MS) medium (2.16 g L^−1^ MS salts [Duchefa Biochemicals; M0221], 0.5% [w/v] Suc, and 8 g L^−1^ phytoagar [Duchefa Biochemicals; P10031], pH to 5.6–5.8, using KOH). Aseptic culture was done under a long-day regime (16/8 h light/dark) in growth cabinets. *Nicotiana benthamiana* plants were grown on a steamed (sterilized for 3 h at 90°C) soil mixture consisting of Einheitserde Classic Kokos (45% [w/w] white peat, 20% [w/w] clay, 15% [w/w] block peat, and 20% [w/w] coco fibers [10-00800-40; Einheitserde Werkverband]).

Seeds for soil-grown plants were germinated after stratification of 4 to 5 d at 4°C in the dark, and plants were grown under standard long-day (16/8 h light/dark) or short-day (8/16 h light/dark) greenhouse conditions between 18°C and 25°C. For strictly controlled development such as during temperature-shift experiments, plants were grown either in growth cabinets (AR-66L2 and AR-66L3; Percival Scientific, CLF PlantClimatics) or walk-in phyto chambers (Johnson Controls) at a humidity of 60% depending on the requirements and watered with prewarmed or precooled tap water. For experiments needing less stringent environmental conditions, plants were cultivated in the greenhouse.

Transgenic, Basta-resistant plants were selected in solium in the cotyledon stage by spraying 150 mL per tray of a 1:1,000 dilution of Basta (contains 200 g L^−1^ glufosinate-ammonium; Bayer CropScience) in tap water that was repeated three times in a 2-d interval.

For aseptic culture, dry seeds were sterilized with chlorine dioxide gas produced from 75% (w/v) Eau de Javel (Floreal Haagen) and 25% (w/v) HCl. Selective MS medium contained 10 mg L^−1^ dl-phosphinotricin (Basta, glufosinate-ammonium; sc-235254; Santa Cruz Biotechnology), 50 mg L^−1^ kanamycin sulfate, 5.25 mg L^−1^ sulfadiazine sodium salt (Sigma-Aldrich; S6387), or 20 mg L^−1^ hygromycin B (Duchefa Biochemicals; H0192).

Plants used in this study were all in the background of the Columbia-0 accession and either wild-type plants or an ethyl methanesulfonate mutant for *PRT1* (*prt1* HygS or *prt1-1*; a kind gift of Andreas Bachmair, Max F. Perutz Laboratories; Bachmair et al., 1993; Potuschak et al., 1998; Stary et al., 2003).

### Stable Plant Transformation and Selection of Transformants

The binary plant expression vectors were retransformed into *A. tumefaciens GV3101-pMP90RK* (*C58C1 Rif^r^ Gm^r^ Km^r^*; Koncz and Schell, 1986) to obtain a bacterial transformation suspension. The identity of the *A. tumefaciens* strains was verified by back transformation of isolated plasmid into *Escherichia coli* DH5α and at least three independent analytical digestions. All constructs were transformed by a modified version of the floral dip method (Dissmeyer and Schnittger, 2011).

Individual T1 (generation 1 after transformation) transgenic plant lines were preselected with Basta or kanamycin as described above. To exclude lines showing position effects (e.g. by disrupting essential genes by the construct T-DNA), the number of insertion loci was determined in a segregation analysis in the T2 generation, and only transgenic plants carrying a single insertion locus were further used. Standard lines were established by isolating T3 plants homozygous for the transgene. Independent representative reference lines displaying a typical conditional phenotype upon temperature upshifts and downshifts were used in the final experiments. Standard lines were established by isolating T3 plants homozygous for the transgene. In order to identify responsive transgenic lines, we prescreened by developmental and histological phenotype.

### Transient Transfection of *N. benthamiana*

For transient transformation of *N. benthamiana*, leaves of 5-week-old plants were infiltrated with the *A. tumefaciens* strain GV3101-pMP90RK (Koncz and Schell, 1986) carrying binary plant expression vectors (Pusch et al., 2011). Agrobacteria were grown to the stationary phase overnight in 10 mL of liquid yeast extract and beef medium and pelleted at 5,000*g* at room temperature. The pellet was washed once in 10 mL of infiltration buffer (10 mm MES, pH 5.6, 10 mm MgSO_4_, and 100 µm acetosyringone; D134406; Sigma-Aldrich) and subsequently resuspended in 10 mL of the same buffer. Agrobacteria strains were always coinfiltrated together with the *A. tumefaciens* strain GV3101 expressing *p19* of the *Tomato bushy stunt virus* from the vector *pBIN6Ip19* to suppress posttranscriptional gene silencing and increase ectopic gene expression (Pusch et al., 2011). Prior to infiltration, bacterial suspensions were adjusted to an OD_600_ of 0.5. Bacterial suspensions containing the lt-BAR transgene were mixed with the *p19* bacteria suspension in a 1:1 ratio (final OD_600_ = 0.5) and used for transformation. Bacterial suspensions were then infiltrated into the epidermis on the lower side of the *N. benthamiana* leaf. Infiltrated areas were marked on the upper side of the leaf with a permanent marker. For an easier infiltration procedure, plants were watered and transferred to standard greenhouse long-day conditions the day before in order to allow plant stomata to open. To allow efficient transformation and expression, plants were kept for 48 h in the greenhouse, before applying temperature-shift experiments by transferring them into a growth cabinet at either permissive or restrictive growth conditions. For one data point, 15 to 20 leaf discs of 5 mm diameter were harvested from infiltrated areas and snap frozen in liquid nitrogen. Extraction was performed using radioimmunoprecipitation assay buffer as mentioned below.

### Microscopy and Documentation of Plants

Photographs of in vitro cultures and histological staining were taken with a stereomicroscope (Stemi 2000-C) equipped with a Zeiss CL 6000 LED illumination unit, and a video adapter 60 C including an AxioCam ERc 5s digital camera (all from Carl Zeiss MicroImaging). Trichome visualization through polarized light microscopy was carried out as described previously (Gudesblat et al., 2012; Pomeranz et al., 2013) with the only change that destaining was achieved with a saturated chloral hydrate solution (dissolve 1 kg in 400 mL of water). Trichomes were visualized using a Nikon AZ100 zoom microscope equipped with a Nikon DS-Ri2 color camera. Microscopy images of trichomes and DAPI-stained nuclei were obtained using the AxioImager system (Zeiss) equipped with two cameras (AxioCam MRm and AxioCam MRc5).

### Protein Extraction and Western-Blot Analysis

Plant tissue (Arabidopsis, leaves or seedlings; *N. benthamiana*, five leaf discs) was collected in a standard 2-mL reaction tube containing three Nirosta stainless steel beads (3.175 mm; 75306; Mühlmeier), snap frozen in liquid N_2_, and stored at −80°C. Material was ground frozen using a bead mill (Retsch; MM400; 45 s, 30 Hz) in collection microtube blocks (adaptor set from TissueLyser II; 69984, Qiagen; Faden et al., 2016b; Dissmeyer, 2017). Tissue was lysed using radioimmunoprecipitation assay buffer (50 mm Tris-Cl, pH 8, 120 mm NaCl, 20 mm NaF, 1 mm EDTA, 6 mm EGTA, 1 mm benzamidine hydrochloride, 15 mm Na_4_P_2_O_7_, and 1% [v/v] Nonidet P-40 supplemented with freshly added EDTA-free Complete Protease Inhibitor cocktail [Roche]). The protein content of the samples was determined using a DirectDetect infrared spectrophotometer (Merck Millipore). Equal protein amounts were resolved by 12% SDS-PAGE. Blotting was done onto a polyvinylidene fluoride transfer membrane by semidry blot using a Trans-Blot SD semidry electrophoretic transfer cell (170-3940; Bio-Rad).

Equal loading and general protein abundance were confirmed by staining of the blotted and probed membranes after immunostaining with Coomassie Brilliant Blue G250. Immunodetection was done using mouse monoclonal anti-HA epitope tag antibody (HA.11, clone 16B12 [MMS-101R; Covance]; 1:1,000) and horseradish peroxidase-conjugated secondary antibodies that were detected by enhanced chemiluminescence using ECL SuperSignal West Pico or Femto (34087 or 34096; Pierce) followed by exposure on autoradiography film. Western-blot data were confirmed by analysis of at least three biological replicates.

### Transcript Analysis

Two-week-old seedlings were collected and snap frozen. After milling, about 50 mg of tissue was used for RNA extraction with the RNeasy Plant Mini Kit (Qiagen). RNA was measured with a spectrophotometer, and quality was assessed via agarose gel electrophoresis. For first-strand cDNA synthesis, 500 ng of total RNA was used with an equimolar mixture of four oligo(dT) primers (CDSIII-*Not*IA/C/G/T; Faden et al., 2016b) and RevertAid H Minus Reverse Transcriptase (Thermo Scientific). One microliter of cDNA was used for PCR analysis using self-made *Taq* in two reactions per sample: one with generic degron-specific primers (DHFR_frw/DHFR_rev) to test transcript levels of the transgene and one with intron-spanning primers EF1ss/EF1as for *ELONGATION-FACTOR1* (*EF1*) as a housekeeping gene (amplicon sizes: genomic, 810 bp; cDNA, 709 bp). Both PCRs were run for 30 cycles.

### Accession Numbers

The following materials are available in public repositories: vector *pEN-L1-K2-L2* (Addgene ID 80684), vector *pAM-PAT*:*ProTRY*:*GW* (Addgene ID 79755), germplasm *prt1-1* (in the background of hygromycin-resistant line 4H prt1; TAIR stock CS119), and pPAM (GenBank accession no. AY027531). Accession numbers are as follows: pAM-PAT-MCS (GenBank AY436765), TRIPTYCHON (GenBank NM_124699), CAPRICE (National Center for Biotechnology Information reference sequence NM_130205), ENHANCER OF TRY AND CPC1 (GenBank NM_100020), and barnase (UniProtKB P00648 [RNBR_BACAM]).
